# Mining public mass spectrometry data to map the bull sperm membrane-associated proteome

**DOI:** 10.1530/RAF-25-0179

**Published:** 2026-04-01

**Authors:** Erin K Klein, Aleona Swegen, Allan J Gunn, Cyril P Stephen, R John Aitken, Zamira Gibb, David A Skerrett-Byrne

**Affiliations:** ^1^ Infertility and Reproduction Research Program, Hunter Medical Research Institute, New Lambton Heights, New South Wales, Australia; ^2^ Centre for Reproductive Science, School of Environmental and Life Sciences, College of Engineering, Science and Environment, University of Newcastle, Callaghan, New South Wales, Australia; ^3^ School of Agricultural, Environmental and Veterinary Sciences, Charles Sturt University, Wagga Wagga, New South Wales, Australia; ^4^ Gulbali Institute for Land, Water and The Environment, Charles Sturt University, Wagga Wagga, New South Wales, Australia; ^5^ Department of Population Health and Reproduction, School of Veterinary Medicine, University of California Davis, Davis, California, USA; ^6^ School of Biomedical Sciences and Pharmacy, College of Health, Medicine and Wellbeing, University of Newcastle, Callaghan, New South Wales, Australia; ^7^ Institute of Experimental Genetics, Helmholtz Zentrum München, German Research Center for Environmental Health Neuherberg, Neuherberg, Germany; ^8^ German Center for Diabetes Research (DZD) Neuherberg, Neuherberg, Germany

**Keywords:** sperm, sperm membrane, bovine, proteome, bioinformatics

## Abstract

**Abstract:**

The sperm plasma membrane is of the utmost importance to the structural integrity and fertilising ability of the spermatozoon. Without a functional membrane, a spermatozoon cannot be expected to survive the arduous journey from testes to oviduct, particularly when its journey is disrupted by assisted reproductive technology (ART). In this study, we endeavoured to characterise a more complete bull sperm membrane-associated proteome through reanalysing publicly available RAW mass spectrometer data files from three previously published studies. Individually, these studies have utilised differing software platforms and inclusion criteria for their identification of membrane-associated proteins. The reanalysis of these datasets with standardised search criteria against up-to-date databases has identified 742 proteins. Amongst the proteins identified, 378 overlap with those reported in earlier studies, whereas we describe 364 previously unreported bovine sperm membrane-associated proteins linked to key functions, including zona pellucida binding. Next, the bovine proteome was mapped to human homologues (humanised), with a 96.8% conversion rate, allowing us to perform a more advanced functional analysis of the identified membrane-associated proteins, highlighting roles in energy metabolism, sperm motility, actin polymerisation, and protein ubiquitination. This deeper understanding of the bull sperm membrane-associated proteome will inform biomarker discovery for evaluating bull fertility and guide strategies to preserve sperm integrity during storage and ART procedures.

**Lay summary:**

In this study, we combined and reanalysed publicly available data files from three previously published studies to generate an updated list of the proteins associated with the membrane of bull sperm cells. We identified 742 proteins in total, of which 364 were not reported previously in the original studies. Membrane proteins are important because they sit on the surface of the sperm and control how it recognises and communicates with the egg. Our functional analysis of these proteins revealed roles in sperm motility, energy production, and key fertilisation events, such as binding to the egg. This study highlights the value of depositing data in public repositories so that older datasets can be reanalysed as analytical tools improve. A greater understanding of sperm membrane-associated proteins will improve knowledge of bull fertility with important implications for breeding and livestock production industries.

## Introduction

While the genetic contribution of a spermatozoon is safely housed within the sperm head until fusion with the egg, there is a prolonged passage through male and female reproductive tracts that the spermatozoon must endure to reach the site of fertilisation (Hermo *et al.* 2010*a*,*b*, Nixon *et al.* 2020). Crucial to the success of this journey is the sperm membrane, protecting the integrity of the cell and maintaining fertilising ability. When spermatozoa are used for artificial insemination (AI) or *in vitro* fertilisation, additional insults to the sperm membrane are imposed by a substantially increased delay between ejaculation and fertilisation, along with the mechanical injury to membranes caused by *in vitro* manipulations, such as cryopreservation (Blässe *et al.* 2012, Gürler *et al.* 2016) and/or flow cytometric sex-sorting (Vishwanath & Moreno 2018, Camara Pirez *et al.* 2020).

Upon epididymal maturation, the sperm membrane is endowed with all the proteins (Skerrett-Byrne *et al.* 2022); it requires to be capable of undergoing remodelling (Aitken *et al.* 2007, Hermo *et al.* 2010*c*, Nixon *et al.* 2020) and triggering capacitation events (Cross 1998, Aitken & Nixon 2013) at the precise time, as well as gaining entry to the oviduct (Yamaguchi *et al.* 2009, Fujihara *et al.* 2014), binding to the zona pellucida (Barbaux *et al.* 2020, Fujihara *et al.* 2020), and finally fusing with the oolemma (Sutovsky 2009, Matsumura *et al.* 2022) for fertilisation to occur. As these membrane proteins are essential for successful fertilisation, they present promising targets for future use as biomarkers for fertility (Nixon *et al.* 2023).

Previous research into the bovine sperm proteome has focused on the whole cell (Peddinti *et al.* 2008, Park *et al.* 2012, Soggiu *et al.* 2013) or proteins found in bovine seminal plasma (Killian *et al.* 1993, Nauc & Manjunath 2000, Jobim *et al.* 2004, Gwathmey *et al.* 2006), with little focus on isolated cellular components, such as the cell membrane, alone. In what is likely the earliest bovine proteomic study to utilise high-throughput mass spectrometry, Peddinti *et al.* identified over 4,000 proteins, but at that time, less than 15% of the proteins they identified had been previously described in the bovine proteome (Peddinti *et al.* 2008). In the past 15 years, the field has been slow to expand, where in 2025, a PubMed search of the terms ‘bull’, ‘sperm’, and ‘proteome’ only returned 182 results (20 of which are review articles). A limited number of these high-throughput studies focus specifically on bull sperm membrane-associated proteins, with only three articles found to have publicly available mass spectrometer (MS) files: Byrne *et al.* (2012), Kasvandik *et al.* (2015), and Shen *et al.* (2021). In all three articles, different approaches are used for the isolation of membrane-associated proteins using spermatozoa from Brahman or Holstein bulls. Moreover, three different MS processing software tools were utilised, with variations in the thresholds used to ascertain highly confident protein identifications.

Capitalising on advancements in MS processing software and label-free algorithms (Kong *et al.* 2017, Skerrett-Byrne *et al.* 2021*a*, Cox 2023, Pini *et al.* 2025), the aim of this study was to bring together existing datasets for uniform analysis against updated databases, to compile a more complete bull sperm membrane-associated proteome. The combined proteome was then mapped to human homologues to maximise the functional insights into this comprehensive proteome. In characterising the bull sperm membrane-associated proteome, much can be learnt about the needs for supporting the integrity of the sperm cell during the *in vitro* manipulations required for ART procedures and will also aid in future identification of biomarkers of fertility.

## Methods

### Proteomic data source and processing

The RAW MS files corresponding to three proteomic studies focusing on bull sperm membrane-associated proteins (Byrne *et al.* 2012, Kasvandik *et al.* 2015, Shen *et al.* 2021) were downloaded via PRIDE (Tables 1 and 2) (Perez-Riverol *et al.* 2021). Consistent with previous studies (Degryse *et al.* 2018, Skerrett‐Byrne *et al.* 2021*b*,*c*, 2022, Murray *et al.* 2021, Trigg *et al.* 2021, Martin *et al.* 2022, Smyth *et al.* 2022, Staudt *et al.* 2022, Pini *et al.* 2025), database searches of each study’s RAW files were performed using Proteome Discoverer 2.5 (Thermo Fisher Scientific, USA). SEQUEST HT was used to search against the UniProt *Bos* database (191,401 sequences, downloaded 18 June 2022). Highly stringent database searching criteria were utilised; this included up to two missed cleavages, a precursor mass tolerance set to 10 ppm, and a fragment mass tolerance of 0.02 Da (higher stringency than the original studies). Trypsin was designated as the digestion enzyme. Cysteine carbamidomethylation was set as a fixed modification, while acetylation (K, N-terminus), phosphorylation (S,T,Y), and oxidation (M) were designated as dynamic modifications. Interrogation of the corresponding reversed database was also performed to evaluate the false discovery rate (FDR) of peptide identification using Percolator on the basis of *q*-values, which were estimated from the target-decoy search approach. To filter out target peptide spectrum matches over the decoy-peptide spectrum matches, a fixed FDR of 1% was set at the peptide level. The resultant protein list was exported from Proteome Discoverer 2.5 as an Excel file and further refined to include only those IDs with a protein identification of high (i.e. FDR ≤ 0.01) and at least one or more unique peptides.

**Table 1:** Summary of bull sperm membrane proteomes accessed and processed.

Study	PRIDE identifier	PMID	Species
Byrne *et al.* (2012)	PXD000007	23081703	*Bos taurus*
Kasvandik *et al.* (2015)	PXD001096	25603787	*Bos taurus*
Shen *et al.* (2021)	PXD019435	34009990	*Bos taurus*

**Table 2:** Comparison of experimental setup that studies utilised.

Study	Bulls used	Semen collection method	Semen processing and storage temperature	Membrane isolation technique	Sample preparation notes	MS technology	Processing software	Precursors and fragment tolerance
Shen *et al.* (2021)	*Bos taurus* (*n* = 20)	Not disclosed	Sex-sorted and extended with OptiXcell 2, temperature not disclosed	Minute plasma membrane protein isolation and cell fractionation kit (modified)	In-gel digestion and high-pH fractionation	Orbitrap Fusion Lumos	Proteome Discoverer (v2.1)	15 ppm, 0.5 Da
Kasvandik *et al.* (2015)	*Bos taurus* (*n* = 3)	Artificial vagina	Whole ejaculate extended 1:1 with triladyl, maintained at 10°C for 3 h	Surface biotinylation and affinity purification in combination with differential ultracentrifugation	In-solution digestion	LTQ Orbitrap XL	MaxQuant (v1.4.0.8)	Not disclosed
Byrne *et al.* (2012)	*Bos indicus* (*n* = 5)	Electro-ejaculation	Density gradient centrifugation (Percoll), complete EDTA-free protease inhibitor tablets added, room temperature (22°C)	Nitrogen cavitation and differential ultracentrifugation	1D-gel or SCX or unfractionated	QStar Elite, UltrafleXtreme, TripleTOF 5600 system	ProteinPilot (v4.01.1)	Not disclosed

When comparing the proteome generated from our uniform reanalysis with the protein lists reported in the original studies, additional post hoc filtering was applied to the published supplementary tables to ensure consistency and rigour across studies. In particular, only proteins identified with high confidence (protein-level FDR ≤ 0.01, or ‘high’ category in Proteome Discoverer) and ≥1 unique peptide and quantified in at least 80% of biological replicates within each original study were retained for cross-study comparison.

In addition, due to the time span of these studies (2012–2021), during which UniProt accessions were updated or deprecated, we cross-referenced primary gene symbols with the current UniProt accession numbers to ensure accurate matching (Supplementary Table 1 (see section on Supplementary materials given at the end of the article)).

### Membrane isolation approaches in source studies

To account for potential differences in membrane enrichment across the original studies, we reviewed the protein isolation workflows described (Table 2): Byrne *et al.* (2012) utilised density gradient centrifugation followed by nitrogen cavitation and differential ultracentrifugation to enrich membrane fractions; Kasvandik *et al.* (2015) employed density gradient centrifugation, surface biotinylation, and affinity purification in combination with differential ultracentrifugation; Shen *et al.* (2021) applied a commercial minute plasma membrane protein isolation and cell fractionation kit to isolate the total membrane protein fraction, including organelle-associated membranes, without reported subsequent differential centrifugation. Collectively, these approaches reflect membrane enrichment strategies rather than strict plasma-membrane-only isolation, and thus, our analysis captures a membrane-associated proteome, including bona fide surface proteins, as well as proteins associated with sub-membrane structures and organelles.

### UniProt mapping and DAVID analyses

Utilising the UniProt (https://www.uniprot.org/) (Ahmad *et al.* 2025) ID mapping feature, the refined bull sperm membrane-associated proteome was mapped back to UniProt knowledge base to ascertain the reviewed status and level of evidence of each protein (Skerrett-Byrne *et al.* 2021*a*,*b*,*c*, 2022, Smyth *et al.* 2022). Reviewed proteins refer to proteins that have been manually assessed by UniProt, while unreviewed proteins are of high quality computationally predicted by TrEMBL. UniProt protein evidence indicates the classification of the type of manually curated evidence supporting a protein's existence, including i) experimental evidence at protein level, ii) experimental evidence at transcript level, iii) protein inferred from homology, iv) protein predicted, and v) protein uncertain. The resulting output was exported to Excel, and a summary of these findings was graphed using GraphPad Prism (version 9.4.1).

The UniProt accession numbers of the refined bull sperm membrane-associated proteome were submitted to DAVID (version 2022q4) (Huang *et al.* 2009), and ‘biological process’, ‘molecular function’, and ‘cellular component’ outputs were exported to Excel. Each gene ontology output was refined to only those categories that showed statistically significant enrichment (*P*-value ≤0.05).

### Humanisation with OmicsBox and Ingenuity Pathway Analysis

Using a custom workflow on the OmicsBox software (version 2.2.4, BioBam Bioinformatics, Spain) (https://www.biobam.com/omicsbox), the refined list of bull sperm membrane-associated proteins was subjected to a cloud-based BLAST search against the human proteome (Götz *et al.* 2008, Skerrett-Byrne *et al.* 2021*a*, Zhang *et al.* 2022). The humanised output was restricted to an e-value cut-off of 4.07E-10 to ensure that accurate homologues were obtained (96.8% conversion). The humanised bull sperm membrane-associated proteome containing UniProt accessions was subjected to analysis with the Ingenuity Pathway Analysis software package (IPA; Qiagen, Hilden, Germany) as previously described (Degryse *et al.* 2018, Skerrett-Byrne *et al.* 2021*a*,*b*,*c*, 2022, Murray *et al.* 2021, Trigg *et al.* 2021, Martin *et al.* 2022, Smyth *et al.* 2022, Staudt *et al.* 2022, Zhang *et al.* 2022). The mapped list (676/718; 94%) was analysed on the basis of predicted protein subcellular location and classification (other excluded), in addition to canonical pathways and ‘disease and functions’ using the IPA *P*-value enrichment score (Krämer *et al.* 2013). To elucidate the most significant enrichments in our analyses, we applied a stringency criterion of *P*-value ≤0.05 (i.e. −log10 *P*-value of ≥1.3).

## Results

### Proteomic characterisation of the bull sperm membrane

To expand the understanding of the bull sperm membrane, we have sourced the RAW mass spectral data from three publicly available studies (Table 1) and processed these datasets with the latest annotation of the bull proteome coupled with highly stringent proteomic analysis tools, returning a complex membrane-associated proteome comprising 742 proteins (Table 3 and Supplementary Table 1; FDR ≤ 0.01). This bull sperm membrane-associated proteome had an average of 3.7 peptides per protein (3.0 unique peptides) with an average protein sequence coverage of 12.6% (Table 3). In addition, 42 proteins were identified to harbour key fertilisation protein post-translational modifications, either acetylation or phosphorylation (Aitken & Nixon 2013, Nixon *et al.* 2020).

**Table 3:** Summary of bull sperm membrane-associated proteome.

Bull sperm membrane	Values
Total proteins	742
Average peptide/protein	3.8
Average unique peptide/protein	3.0
Average protein coverage (%)	12.6
Proteins with modifications	42

**Figure 1:**
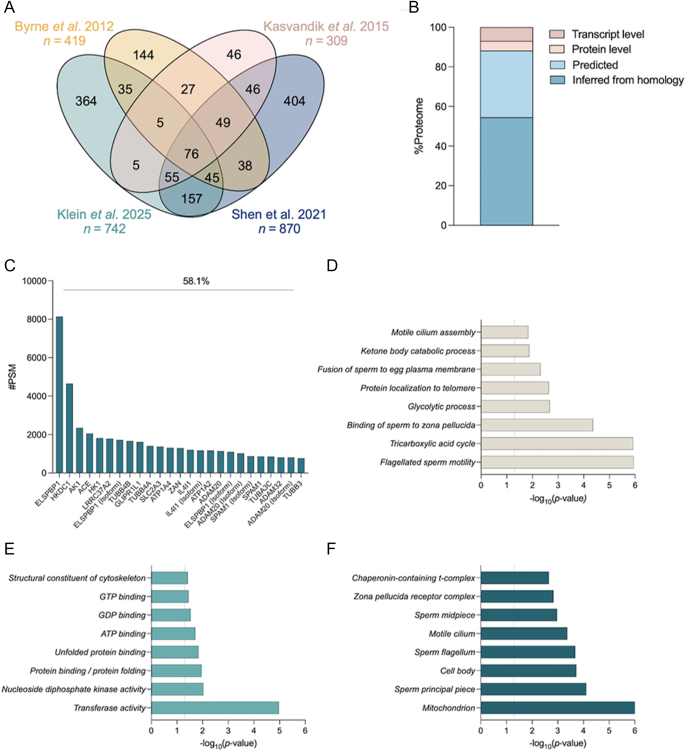
Analysis of the refined bull sperm membrane-associated proteome. (A) Venn diagram overlap of the proteins identified in each of the three studies and our proteome generated from the reanalysis of their RAW mass spectral data files. (B) Using UniProt, the refined bull sperm membrane-associated proteome was assessed to determine the level of supporting evidence for each protein. (C) The peptide spectrum match (PSM) load carried by the 25 most abundant bull sperm membrane-associated proteins accounts for 58.1% of the total PSM load. Gene symbols are displayed on the x-axis. Where gene symbols were lacking or insufficient, later human homology matching was used to assign the most appropriate gene symbol. The refined list (FDR ≤ 0.01) of proteins was subjected to a gene ontology analysis, classifying the most significant (D) biological processes, (E) molecular functions, and (F) cellular compartments. The dotted line represents the threshold for statistical significance (*P*-value ≤0.05; −log10 (*P*-value) ≥1.3).

Importantly, this newly generated proteome was compared to each study’s protein list (see Methods): 309 – Kasvandik *et al.*, 870 – Shen *et al.*, and 419 – Byrne *et al.* (Fig. 1A). An initial comparison of each list individually with the new proteome yielded a 45.6, 41.7, and 38.4% match in Kasvandik *et al.*, Shen *et al.*, and Byrne *et al.*, respectively. The three lists were combined with ours to produce a total proteome of 1,496 proteins, ensuring that any overlapping proteins were accounted for, demonstrating an overlap of 378 proteins (25%), including adenylate kinase isoenzyme 1 (AK1), epididymal sperm-binding protein 1 (ELSPBP1), interleukin 4-induced 1 (IL4I1), and acrosin-binding protein (ACRBP). Applying our stringent criteria to the published datasets uncovered 364 proteins detected exclusively in our analysis. Of these, 231 are novel, while the remaining 133 had been previously reported but were excluded when a rigorous identification criterion was applied, namely retention only of proteins identified with FDR ≤ 0.01 and quantified in at least 80% of biological replicates, as outlined in the Methods section. Amongst the novel proteins include leucine-rich repeat-containing protein 37A (LRRC37A2), polyubiquitin-B (UBB), and disintegrin-and-metalloproteinase domain-containing protein 1a (ADAM1A).

Seeking to interrogate the level of supporting evidence of this new bull sperm membrane-associated proteome, UniProt revealed that only 4.8% (36) of these proteins have proven evidence at the protein level (Fig. 1B). This further breaks down to 6.9% (51) with evidence at the transcript level, 33.7% (250) predicted to exist based on genome annotation, and 54.6% (405) inferred from homology (Fig. 1B). Spermatozoa are dominated by a subset of abundant proteins; with this proteome focused on membrane-associated proteins, we sought to investigate what are the dominating proteins through the peptide spectrum match (PSM) load (Smyth *et al.* 2022). This analysis revealed 25 proteins (3.4% of total proteome) and captured 58.1% of the total PSM load (Fig. 1C; Supplementary Table 2), a reflection of the most dominant proteins across the mass spectrometry analyses. Chief amongst these membrane-associated proteins were important energy kinases (hexokinase and AK1) and, critically, zona pellucida-binding proteins (zonadhesin, disintegrin-and-metalloproteinase domain-containing protein 32 (ADAM32), and hyaluronidase PH-20/sperm adhesion molecule 1 (SPAM1)).

### Gene ontology analysis of bull sperm membrane-associated proteome

An initial gene ontology (GO) enrichment analysis was carried out using DAVID (Huang *et al.* 2009, Sherman *et al.* 2022) on the refined proteome (FDR ≤ 0.01) to gain insight into the biological processes and molecular functions at play, as well as the distribution of cellular components linked to these sperm membrane-associated proteins. These *in silico* analyses revealed significant enrichments (*P*-value ≤0.05) at each of the functional levels assessed. Assessment of the biological processes tying this proteome together included, notably, enrichment of ‘flagellated sperm motility’, ‘binding of sperm to zona pellucida’, ‘glycolytic process’, and ‘protein stabilisation’ (Fig. 1D; Supplementary Table 3). Molecular functions were dominated by enzymatic activity and protein remodelling functions, including ‘transferase activity’, ‘protein binding involved in protein folding’, ‘unfolded protein binding’, and ‘ATP binding’ (Fig. 1E; Supplementary Table 3). Finally, the putative cellular components of the membrane-associated proteins included ‘mitochondrion’, ‘sperm principal piece’, ‘sperm flagellum’, and most notably ‘zona pellucida receptor complex’ (Fig. 1F; Supplementary Table 3).

### Humanisation of bull sperm membrane-associated proteome

**Figure 2:**
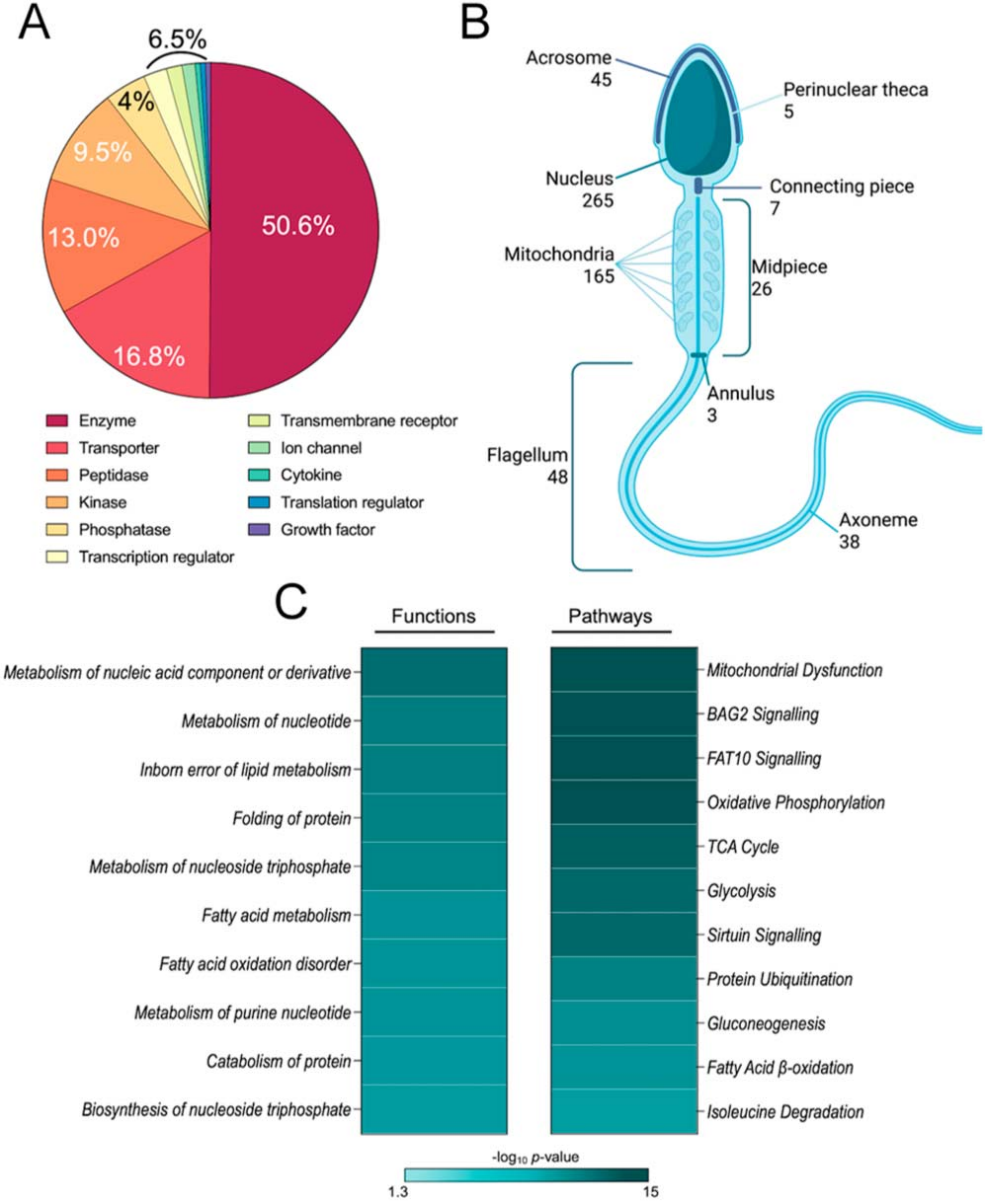
Characterisation of the humanised bull sperm membrane-associated proteome. (A) Classification of protein type and cellular location were determined using Ingenuity Pathway Analysis (IPA) on the refined humanised list (duplicates removed). In both cases of protein type and cellular location, ‘other’ was excluded visually. (B) Analysis of sperm-specific locations using UniProt and EMBL-EBI gene ontology annotations; the locations include acrosome, annulus, axoneme, flagellum, mid-piece, mitochondria, nucleus, and perinuclear theca. Created with Biorender.com. (C) Heat maps of the top 10 most highly enriched (*P* ≤ 0.05) functions and pathways as determined through IPA. Aspects of this figure were created in BioRender. Skerrett-Byrne, DA (2026) (https://BioRender.com/fpi4edu).

Given the poor annotation of the bull proteome with both UniProt and DAVID, we elected to review the human homologues of our bovine proteome to gain greater insights into the composition and functions underpinning the bull sperm membrane-associated proteome. This approach successfully mapped 718 proteins, 96.8% of the total bovine proteins to human protein homologues (Supplementary Table 1). Once duplicates of proteins were removed (e.g. possible isoforms), the remaining humanised proteome (678 proteins) was then subjected to analysis with Ingenuity Pathway Analysis (IPA) to gain a greater understanding of the categorisation of protein types (Fig. 2A). Enzymes (200 proteins: 50.1%) were the most dominant protein type, followed by transporters (67 proteins: 16.8%), peptidases (52 proteins: 13.0%), kinases (38 proteins: 9.5%), and phosphatases (16 proteins: 4.0%) (Fig. 2A). The remaining types of transcription regulator, transmembrane receptor, ion channel, cytokine, translation regulator, and growth factor collectively accounted for 6.5% (26 proteins).

With the bovine proteins now converted to their human homologues, we leveraged UniProt’s rich subcellular annotations to assign each protein to specific sperm compartments, with some proteins mapped to multiple sites (Fig. 2B). This approach showcased that 7.0% of the humanised sperm proteins were mapped to the acrosome (45 proteins) and perinuclear theca (5 proteins). The highest annotated location was the nucleus with 36.9% of the total proteome (265 proteins). Further along the spermatozoon, 7 were mapped to the connecting piece and 26 proteins were annotated to the mid-piece, which also houses 165 mitochondria-related proteins. Finally, 3 proteins were located at the annulus, 48 were part of the flagellum, and 38 were axoneme proteins.

Finally, IPA was used to explore the functional role of these sperm membrane-associated proteins. To achieve this, the dataset was first subjected to downstream function analyses, identifying several significantly enriched (*P*-value ≤0.05) energy metabolism functions, including ‘metabolism of nucleic acid component or derivative’ (1.26E-13), ‘fatty acid metabolism’ (1.10E-10), and ‘synthesis of ATP’ (4.90E-08) (Fig. 2C; Supplementary Table 4), driven by proteins such as AK1 and transforming protein RhoA (RHOA). Additional function enrichments of ‘folding of protein’ (4.79E-12) and ‘cell movement of sperm’ (4.37E-07) were observed. Pathway analysis revealed synergy with the downstream functions with the significant enrichment of ‘glycolysis’ (5.01E-14) and ‘fatty acid β-oxidation’ (1.66E-10) (Fig. 2C; Supplementary Table 4). Moreover, pathway analysis identified significant enrichment of ‘BAG2 signalling’ (2.51E-23), ‘oxidative phosphorylation’ (2.00E-17), and ‘protein ubiquitination pathway’ (5.01E-12), including ATP synthase F1 subunit alpha (ATP5F1A), cytochrome c oxidase subunit 5A (COX5A), and heat shock protein family A (HSP70) members 2, 5, 8, and 9 mapping to these pathways. Additional important sperm-related pathways were found to be significantly enriched, including ‘protein kinase A signalling’ and ‘RHOA signalling’.

## Discussion

The sperm surface membrane is of key importance for its fertilising capacity, undergoing remodelling of the protein and lipid profile during epididymal maturation, which is sustained through to their ascent of the female reproductive tract (Aitken *et al.* 2007, Nixon *et al.* 2019, Skerrett-Byrne *et al.* 2022). To characterise the protein components of the bull sperm membrane and explore their functions regarding fertilisation, we have sourced the proteomic RAW data files (spectral data generated by MS) from three studies focusing on the bull sperm membrane with publicly available data. RAW data can be reprocessed retrospectively, representing a ‘living’ resource that can be utilised against continually improved annotated databases by researchers in the pursuit of new discoveries (Martens & Vizcaíno 2017, Pini *et al.* 2025). From the time of the first study employed here until our reprocessing of the data, the *Bos* database had grown from 70,452 to 191,401 proteins, a 2.7-fold increase. Alongside this expansion in the *Bos*-annotated proteome, a refinement of obsolete/duplicated proteins has removed 21 proteins, which were identified in Byrne *et al.* (2012). These public repositories for RAW MS files are a valuable resource that is currently underutilised. Herein, we have reprocessed all three studies collectively and applied uniform highly stringent parameters with updated annotation of the *Bos* proteome (Ahmad *et al.* 2025). It is important to acknowledge that the datasets analysed here were generated using differing membrane enrichment approaches, rather than protocols designed to isolate pure plasma membrane fractions. As such, the resulting proteome represents a membrane-associated sperm protein landscape, including plasma membrane proteins alongside cytoskeletal, mitochondrial, and organelle-linked proteins that co-purify with membrane fractions. This is consistent with previous sperm membrane proteomic studies (reviewed in (Brewis & Gadella 2010)) and reflects both the complex ultrastructure of the sperm surface and the technical challenges of isolating this compartment without loss of biologically relevant components. Furthermore, UniProt/IPA annotations may not fully reflect sperm-specific localisation, since many proteins can occupy multiple compartments and livestock proteome annotations remain incomplete. In this context, our study provides a harmonised and biologically informative resource based on available enrichment strategies, while also underscoring the need for standardised plasma membrane isolation protocols for future work.

Through this reanalysis of 96 RAW data files, we have added 364 bull sperm membrane-associated proteins, not previously identified. The reanalysis of these combined datasets with our parameters applied generated a list of 742 proteins where only 50.9% had been previously captured within the filtered analyses (378 proteins) (Fig. 1A). Combined with our reanalysis, these studies generated a proteome of 1,496 unique proteins. The largest contributor of these proteins was Shen *et al.* (2021), 870 of the 1,496 total proteins (58.2%) (Fig. 1A); however, it should be noted that much less stringent search criteria were applied in their analysis (precursor mass tolerance = 15 ppm; fragment tolerance = 0.5 Da) compared to that of our own (precursor mass tolerance = 10 ppm; fragment tolerance = 0.02 Da).

Fifteen of the proteins identified in our analysis have been previously identified in bull samples and correlated to bull fertility status (reviewed in Klein *et al.* (2022)), three of which were amongst our top 25 most abundant based on PSM load (Fig. 1C; Supplementary Table 2). AK1 was higher in spermatozoa and seminal plasma from high-fertility bulls (D’Amours *et al.* 2010, Kasimanickam *et al.* 2019), tubulin beta-3 chain (TUBB3) was higher in spermatozoa from high-fertility bulls (Kasimanickam *et al.* 2019), and epididymal sperm-binding protein 1 (ELSPBP1) was higher in spermatozoa and seminal plasma from low-fertility bulls (D’Amours *et al.* 2010, Kasimanickam *et al.* 2019). In addition, 50 of the identified proteins have been previously correlated to high or low motility, as separated by density gradient centrifugation (D’Amours *et al.* 2018). Seven of these were amongst the top 25 most abundant proteins in the bull sperm membrane-associated proteome based on PSM (Fig. 1C; Supplementary Table 2); ADAM32, AK1, hexokinase-1 (HK1), SPAM1, and IL4I1 were all found in higher abundance in high-density (high-motility) spermatozoa, while ELSPBP1 and angiotensin-converting enzyme (ACE) were found in higher abundance in low-density (low-motility) spermatozoa (D’Amours *et al.* 2018).

In keeping with the scope of the published studies (Byrne *et al.* 2012, Kasvandik *et al.* 2015, Shen *et al.* 2021), in which the samples had not been differentiated based on bull fertility status, we are unable to draw direct correlations between identified proteins and fertilising ability in this study. However, the functions of many identified proteins have previously been associated with various aspects of fertility. Amongst the novel proteins identified in our reanalysis were important actin polymerisation proteins, such as dynein axonemal intermediate chain 3 (DNAI3) (Zhang *et al.* 2018), and sperm acrosome-associated protein 9 (SPACA9) (Gui *et al.* 2022), critical for those remodelling events preceding induction of acrosome reaction (Skerrett-Byrne *et al.* 2022). We noted several proteolysis-capable proteins, including disintegrin-and-metalloproteinase domain-containing proteins (ADAMs) 9, 18, and 29 (Blobel *et al.* 1992, Moreno *et al.* 2011), as well as transmembrane protease serine 9 (TMPRSS9) and putative serine protease 4 (PRSS42P) (Yoneda *et al.* 2013). In addition, we detected capacitation-related protein, maestro heat-like repeat-containing protein family member 2B (MROH2B) (Stanger *et al.* 2016). To facilitate cross-species comparison, all these identifications have been queried in the ShinySpermKingdom app (Pini *et al.* 2025), revealing that DNAI3 and SPACA9 are broadly conserved across diverse vertebrate groups (mammals, birds, and even reptiles), with only a few sporadic absences, such as MROH2B, PRSS42P, ADAM proteins 9, 18, and 29, being largely confined to placental mammals. Intriguingly, TMPRSS9 emerges as a bull-exclusive membrane protease, hinting at a bovine-specific adaptation, thereby adding further credence to these novel proteins and providing the field with an interactive resource for investigating their roles in sperm function.

Through GO enrichment analysis of the bull sperm membrane-associated proteome, expected biological processes related to fertilising ability were noted, including ‘binding of sperm to zona pellucida’ and ‘fusion of sperm to egg plasma membrane’ (Fig. 1D; Supplementary Table 3). Many of the biological processes and molecular functions revealed by the analysis were related to enzymatic activity or in support of energy production. However, other biological processes related to ‘protein stabilisation’ (Fig. 1D; Supplementary Table 3) and molecular functions in ‘protein binding involved in protein folding’ and ‘unfolded protein binding’ (Fig. 1E; Supplementary Table 3) highlight the importance of protein remodelling events in the sperm membrane for producing a functionally competent spermatozoon (Skerrett-Byrne *et al.* 2022). In addition, the GO analysis was used to identify the location where a function is carried out (cellular component). For the bull sperm membrane-associated proteome, these included ‘mitochondrion’, ‘sperm principal piece’, ‘sperm flagellum’, and ‘zona pellucida receptor complex’ (Fig. 1F; Supplementary Table 3). While ‘zona pellucida receptor complex’ is an expected and encouraging result, locations in the mitochondria and flagellum could be from proteins with functions in more than one location or suggest incomplete isolation of membrane proteins in the original samples.

Unsurprisingly, research on bovine proteins is limited in comparison with rodent models and humans. From the generated bull sperm membrane-associated proteome, UniProt revealed that only 4.8% (36) of the identified proteins have proven evidence at the protein level (Fig. 1B). In view of this, to extract more information from the dataset produced, we utilised the OmicsBox software to humanise our proteome (Skerrett-Byrne *et al.* 2021*a*, Zhang *et al.* 2022). Annotation of the bull proteome is considerably behind that of the human proteome, but these proteins are highly conserved across species as reflected in successful BLAST mapping to human homologues, with 96.8% of the original proteome maintained. Notably, of the bovine accessions identified in this study, only 4.2% of the proteins had been manually reviewed, compared with 100% of the human accessions. This demonstrates the comparative depth of knowledge of the human proteome, affording us access to more powerful bioinformatics tools for characterising the location, pathways, and functions of these sperm membrane proteins (Pini *et al.* 2025). The humanised proteome (678 proteins) was subjected to analysis with IPA to categorise proteins by type, with enzymes accounting for the majority (200 proteins; 50.1%) (Fig. 2A).

One of the advantages this approach offered was UniProt annotation of cellular location, allowing us to build on human findings to map sperm-specific locations of proteins across important membrane remodelling regions, including the acrosome and perinuclear theca (Zhang *et al.* 2022) (Fig. 2B). This level of annotation will greatly progress bovine sperm research, fast tracking protein selection for researchers to pursue. Taking advantage of these human homologues, we could further interrogate our dataset against IPA, one of the most comprehensive, manually curated tools to explore the biological context of large datasets (Krämer *et al.* 2013). This approach revealed an evident importance of these sperm membrane-associated proteins in driving functions related to energy production and metabolism, reflected in the significant enrichment of proteins associated with ‘fatty acid metabolism’ and ‘synthesis of ATP’ (Fig. 2C; Supplementary Table 4). These processes contribute to sperm motility as they ascend the female reproductive tract (Islam *et al.* 2021), a finding supported by the significant enrichment of proteins associated with ‘cell movement of sperm’ in the present study. Additional energy-demanding processes are involved in sperm membrane remodelling during capacitation; we found significant enrichment of ‘protein kinase A (PKA) signalling’ pathway proteins, critical for capacitation and a driver of acrosomal remodelling (Lefièvre *et al.* 2002, Battistone *et al.* 2014),and ‘RHOA signalling’, which regulates actin polymerisation (Skerrett-Byrne *et al.* 2022), a key step in the acrosome reaction. Notably, amongst our top enriched pathways was ‘protein ubiquitination’, a protein post-translational modification implicated in sperm proteasome function to ensure penetration of the zona pellucida (Sawada *et al.* 2002, Sakai *et al.* 2004, Sutovsky *et al.* 2004).

**Figure 3:**
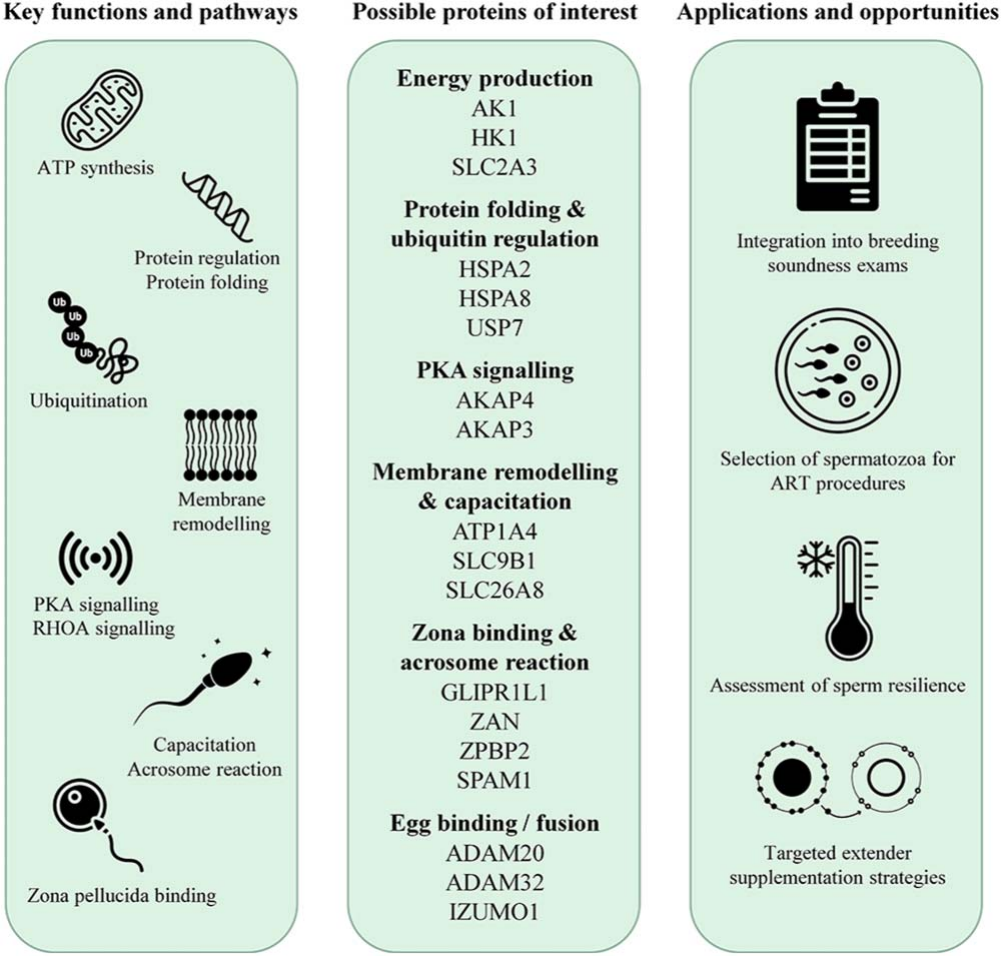
Conceptual schematic linking bovine sperm membrane-associated proteins to functional pathways and translational applications. Illustrative overview of the principal functions and pathways identified in the bovine sperm membrane-associated proteome, highlighting roles in key fertilisation-related processes. Selected proteins are emphasised for future consideration as potential biomarkers of bull fertility, as their expression and functional activity may correlate with sperm performance and fertilisation success. These associations underscore their opportunity for use in fertility assessments and may inform strategies to optimise sperm evaluation, selection, preservation, and functional support during ART.

Harmonised reanalysis of datasets seeks to reduce analytical bias and improve cross-study comparability, strengthening confidence in protein identification (Pini *et al.* 2025). This approach reveals newly identified sperm membrane-associated proteins, expanding insight into key sperm functional pathways, including those associated with energy metabolism and mitochondrial function, cytoskeletal and actin remodelling, protein stabilisation and ubiquitination, and zona pellucida-binding and fertilisation processes. By increasing proteomic depth, lower abundant but functionally relevant proteins associated with sperm competence may be identified. The creation of an expanded sperm membrane-associated proteome substantially broadens the candidate pool for biomarker discovery, providing a more comprehensive foundation for fertility assessment and refinement of bull breeding soundness examinations. These findings also have implications for ART, where molecular signatures associated with resilience could inform sire selection for semen destined for sex-sorting or cryopreservation. Identifying proteins linked to oxidative stress resistance, membrane stabilisation, and capacitation may enable prediction of individuals better suited to withstand the mechanical, thermal, and osmotic stresses imposed during semen processing. Furthermore, these insights may inform the development of extender formulations, including antioxidants and membrane-stabilising factors designed to mitigate reactive oxygen species-mediated damage and preserve functional integrity. These opportunities are summarised schematically in Fig. 3.

Importantly, the translational value of candidate biomarkers will depend on determining whether key proteins are conserved across cattle breeds. Although fertility differences exist between *Bos taurus* and *Bos indicus* cattle, they are frequently attributed to physiological traits (reviewed in Sartori *et al.* (2010)) rather than clearly defined molecular disparities. Typically, lower fertility in *Bos indicus* breeds has been linked to female traits, including later puberty, shorter duration of oestrous behaviour, and longer postpartum anoestrus (Pinheiro *et al.* 1998, Abeygunawardena & Dematawewa 2004, Nogueira 2004). However, studies suggest that there could be bull differences related to seminal plasma content and sperm resilience (Nichi *et al.* 2006, DasGupta *et al.* 2022). Establishing whether fertility-associated sperm proteins are conserved, differentially expressed, or uniquely regulated across subspecies will therefore be critical for ensuring broad applicability of identified biomarkers. Integration of expanded proteomic datasets with physiological and genomic data represents a necessary next step toward precision sire selection and improved reproductive efficiency.

In conclusion, we have demonstrated the utility of *in silico* analysis of publicly available MS data to further characterise the protein content of the mature bull sperm membrane. This analysis revealed proteins with key roles in energy production, zona pellucida binding, and protein stabilisation and remodelling, demonstrating the crucial functions of the sperm membrane in maintaining fertilising ability for the spermatozoon. These new insights derived from previously published datasets demonstrate the value of providing data to public depositories to progress the field. The resources from this study provide a strong platform from which future studies can build to tease apart the functional importance of the novel proteins identified, without prior sperm annotation, and generate a panel of sperm selection markers for high-quality spermatozoa important for AI programmes.

## Supplementary materials









## Declaration of interest

DA Skerrett-Byrne is an Associate Editor of *Reproduction & Fertility* and was not involved in the review or editorial process for this paper, on which he is listed as an author. The authors declare no conflicts of interest.

## Funding

This work was supported by funding from Meat and Livestock Australia (Grant B.GBP.0030) awarded to ZG, AS, AJG, and CPS. AS was the recipient of an Australian Research Council (ARC) Discovery Early Career Researcher Award (DECRA) (DE220100121). ZG was the recipient of an ARC Future Fellowship (FT220100557).

## Author contribution statement

EKK and DAS-B conceived the study, designed the methodology, performed investigation and visualisation, and wrote the original draft of the manuscript; ZG and DAS-B acquired resources; EKK, AS, AJG, CPS, RJA, ZG, and DAS-B reviewed and edited the manuscript; ZG acquired funding; and AS, ZG, and DAS-B supervised the study.

## Data availability

All RAW mass spectral data utilised in this study can be obtained via PRIDE using the identifiers listed in Table 1.
